# Reduced total serum bilirubin levels are associated with ulcerative colitis

**DOI:** 10.1371/journal.pone.0179267

**Published:** 2017-06-08

**Authors:** Kathleen M. Schieffer, Shannon M. Bruffy, Richard Rauscher, Walter A. Koltun, Gregory S. Yochum, Carla J. Gallagher

**Affiliations:** 1 Department of Surgery, Division of Colon and Rectal Surgery, The Pennsylvania State University College of Medicine, Hershey, Pennsylvania, United States of America; 2 Center for Clinical and Translational Research, Virginia Commonwealth University, Richmond, Virginia, United States of America; 3 Department of Public Health Sciences, The Pennsylvania State University College of Medicine, Hershey, Pennsylvania, United States of America; 4 Department of Biochemistry & Molecular Biology, The Pennsylvania State University College of Medicine, Hershey, Pennsylvania, United States of America; 5 Department of Chemistry and Physics, Lincoln University, Lincoln University, Pennsylvania, United States of America; University Hospital Llandough, UNITED KINGDOM

## Abstract

Chronic inflammation associated with inflammatory bowel disease (IBD) results in increased oxidative stress that damages the colonic microenvironment. Low levels of serum bilirubin, an endogenous antioxidant, have been associated with increased risk for Crohn’s disease (CD). Therefore, the aim of this study was to examine whether total serum bilirubin levels are associated with ulcerative colitis (UC). We identified a retrospective case-control population (*n* = 6,649) from a single tertiary care center, Penn State Hershey Medical Center (PSU) and a validation cohort (*n* = 1,996) from Virginia Commonwealth University Medical Center (VCU). Cases were age- and sex-matched to controls (PSU: CD *n* = 254, UC *n* = 187; VCU: CD *n* = 233, UC *n* = 124). Total serum bilirubin levels were obtained from de-identified medical records and segregated into quartiles. Logistic regression analysis was performed on each quartile of total serum bilirubin compared to the last quartile (highest bilirubin levels) to determine the association of total serum bilirubin with UC. Similar to CD patients, UC patients demonstrated reduced levels of total serum bilirubin compared to controls at PSU and VCU. The lowest quartile of total serum bilirubin was independently associated with UC for the PSU (OR: 1.98 [95% CI: 1.09–3.63]) and VCU cohorts (OR: 6.07 [95% CI: 3.01–12.75]). Lower levels of the antioxidant bilirubin may reduce the capability of UC patients to remove reactive oxygen species leading to an increase in intestinal injury. Therapeutics that reduce oxidative stress may be beneficial for these patients.

## Introduction

Inflammatory bowel diseases (IBD), which include Crohn’s disease (CD) and ulcerative colitis (UC), are idiopathic diseases involving chronic inflammation of the gastrointestinal tract. Although not well understood, the pathogenesis of IBD is thought to result from an interplay of genetic predisposition, environmental factors, microbial dysbiosis, and immunological dysregulation [1]. Chronic inflammation is associated with increased oxidative stress, which can be detrimental to the colonic microenvironment. Therefore, it is necessary to understand why IBD patients are at higher risk for increased oxidative stress in order to determine whether reactive oxygen species can be pharmacologically mitigated to prevent the onset or to treat IBD-affected individuals [2].

A previous study found that reduced levels of bilirubin may influence risk of CD development [3]. Bilirubin is a potent endogenous antioxidant that protects against lipid peroxidation [4, 5]. IBD patients with reduced antioxidant capacity display increased levels of lipid peroxidation markers such as serum thiobarbituric acid reactive substances (TBARS) [6]. In the liver, bilirubin is conjugated to glucuronic acid by uridine diphospho-glucuronosyltransferase 1A1 (UGT1A1) to form the water-soluble bilirubin diglucuronide, also known as conjugated bilirubin. Once metabolized, conjugated bilirubin enters the gallbladder where it becomes a constituent of the bile contents. Bile enters the intestines and the conjugated bilirubin undergoes further metabolism where it can either re-enter circulation through the portal vein for urinary excretion or undergo secondary metabolism by intestinal bacteria to stercobilin for fecal excretion. UGT1A1 is the only enzyme capable of converting bilirubin to the water-soluble bilirubin diglucuronide, preparing it for excretion. Although the role of serum bilirubin in CD has been evaluated, the association of bilirubin and UC is not well understood. This study proposes that like CD, UC patients demonstrate lower serum bilirubin levels compared to non-IBD controls. To test this hypothesis, a large case-control study of UC patients and controls was performed on patients from Penn State Hershey Medical Center and a validation cohort from Virginia Commonwealth University Medical Center.

## Materials and methods

### Control and IBD patient populations

Subjects were obtained from the National Institutes of Health-supported Informatics for Integrating Biology and the Bedside (i2b2) database [7] at the Penn State Hershey Medical Center (PSU). This is a database that de-identifies electronic medical records for all patients seen at Penn State Hershey Medical Center from January 1, 2011 until the present date and is updated monthly. We elected to analyze only Caucasians as this race is the most abundantly represented in our study population. Moreover, it is well established that serum bilirubin levels differ between races [8]. If additional races were included, we would introduce a confounding variable into our study. To define our control population (*n* = 6,169), the database was queried by searching all Caucasians with a total serum bilirubin test performed from January 1, 2011 to October 1, 2014 and during the same financial encounter as a routine medical exam to avoid bias for lab tests ordered due to co-morbidity. Control patients were excluded if they had a history of diseases known to influence bilirubin levels, including: liver disease, other intestinal diseases, biliary disease, gallbladder disease, hemolytic anemia, hemochromatosis, benign and malignant neoplasms of colon, small intestine, liver, and gallbladder, and IBD. The IBD patient population was established by querying the database for Caucasians who were diagnosed with CD (*n* = 273) or UC (*n* = 207) and had a total serum bilirubin test performed. Individuals were excluded using the same criteria as controls but omitting IBD. As a validation cohort for this study, the same queries were performed using the i2b2 database at Virginia Commonwealth University (VCU), obtaining data from patients seen at VCU Medical Center from January 1, 2007 to June 1, 2015. Overall, a total of 1,565 controls, 289 CD patients, and 142 UC patients were identified for our study population. This study was determined to be exempt by the PSU Institutional Review Board and the exempt status was reciprocated by the VCU Institutional Review Board.

### Statistical analysis

Bilirubin association from i2b2 was performed by taking the average of total serum bilirubin tests for individuals with more than one lab value. From the distribution, individuals found in the top and bottom 2% of the total distribution were excluded to account for potential confounding variables missing in the i2b2 database, such as smoking status, medical therapy, and UGT1A1*28 genotype. However, analysis of the entire distribution yielded similar trends. Data was stratified by age and sex. Cases were randomly matched to controls by exact 1:1 matching of age and sex using the *MatchIt* package [9, 10] in R version 3.3.3 (The R Project for Statistical Computing, Vienna, Austria). Wilcoxen Rank Sum test was performed on non-transformed data using R version 3.3.3. Logistic regression was performed to evaluate the association between total serum bilirubin levels and IBD. Bilirubin was divided into quartiles using the 25^th^, 50^th^, and 75^th^ percentiles. Logistic regression was performed for each quartile, using the last quartile (highest bilirubin values) as the reference since this was most representative of the control population in R version 3.3.3. Logistic regression was adjusted for age and sex.

## Results

### Total serum bilirubin levels are reduced in IBD patients

Using the i2b2 database, case and control populations were identified for CD and UC to investigate whether total serum bilirubin levels differ between Caucasian IBD and non-IBD controls. Total serum bilirubin values were obtained from patients between January 1, 2011 to October 1, 2014 and for patients with multiple lab values, their results were averaged. A total serum bilirubin test was performed in controls during a routine medical exam and individuals were excluded from the cohort if they had a history of diseases that could affect bilirubin levels. The reference range for total serum bilirubin at PSU is 0.2–1.1 mg/dL. Cases were 1:1 age- and sex-matched to controls, resulting in a population of *n* = 254 for the CD analysis and *n* = 187 for the UC analysis. Overall, both CD and UC patients demonstrated lower serum bilirubin levels compared to the non-IBD control population (CD *P* <0.0001 and UC *P* = 0.0003) (Table 1). The same trends were seen when the data was stratified by age (4 out of 8 groups) and when the full data set was analyzed (S1 Table).

**Table 1:** Comparison of total serum bilirubin between age- and sex-matched inflammatory bowel disease patients and controls at the Penn State Hershey Medical Center.

**Crohn’s Disease**					
**Age Group**	**CD (*n*)**	**CD Bilirubin (mg/dL)** **Median (IQR)**	**Controls (*n*)**	**Control Bilirubin (mg/dL)** **Median (IQR)**	***P*-value**
<20	13	0.40 (0.35–0.50)	13	0.50 (0.30–0.85)	0.501
20–39	98	0.50 (0.40–0.65)	98	0.60 (0.43–0.80)	**0.002**
40–59	90	0.50 (0.40–0.70)	90	0.60 (0.50–0.80)	**0.006**
≥60	53	0.60 (0.45–0.70)	53	0.60 (0.50–0.80)	0.248
Overall	254	0.50 (0.40–0.68)	254	0.60 (0.48–0.80)	**<0.0001**
**Ulcerative Colitis**					
**Age Group**	**UC (*n*)**	**UC Bilirubin (mg/dL)** **Median (IQR)**	**Controls (*n*)**	**Control Bilirubin (mg/dL)** **Median (IQR)**	***P*-value**
<20	6	0.33 (0.25–0.35)	6	0.45 (0.40–0.70)	0.077
20–39	59	0.50 (0.40–0.62)	59	0.60 (0.50–0.75)	**0.009**
40–59	71	0.50 (0.40–0.64)	71	0.60 (0.45–0.75)	**0.008**
≥60	51	0.60 (0.40–0.73)	51	0.67 (0.55–0.80)	0.084
Overall	187	0.50 (0.40–0.70)	187	0.60 (0.50–0.80)	**0.0003**

Data collected from electronic medical records at Hershey Medical Center from 2011–2014. Median and interquartile range (IQR) were calculated. *P*-value performed by Wilcoxen Rank Sum test.

### Low levels of total serum bilirubin are associated with IBD

To measure the association of total serum bilirubin and IBD, total serum bilirubin levels were segregated into quartiles (≤0.50 mg/dL, 0.51–0.60 mg/dL, 0.61–0.80 mg/dL, and ≥0.81 mg/dL) and logistic regression was performed for each quartile, using the last quartile or highest bilirubin values as the reference group since this is the best reflection of the control population. For both CD and UC, total serum bilirubin levels were independently associated with IBD (Table 2). For patients in the lowest quartile of total serum bilirubin, logistic regression analysis identified a significant association with both CD (OR: 1.91 [95% CI: 1.26–2.91]) and UC (OR: 1.98 [95% CI: 1.09–3.63]) in the matched analysis and for the full data set (S2 Table).

**Table 2:** Odds of Crohn’s disease and ulcerative colitis by total serum bilirubin in the Penn State Hershey Medical Center cohort.

Total Serum Bilirubin	Crohn’s Disease OR (95% CI)	Ulcerative Colitis OR (95% CI)
≤0.50 mg/dL	1.91 (1.26–2.91)	1.98 (1.09–3.63)
0.51–0.60 mg/dL	1.00 (0.62–1.63)	0.84 (0.42–1.69)
0.61–0.80 mg/dL	1.11 (0.69–1.79)	0.78 (0.40–1.55)
≥0.81 mg/dL	Reference	Reference

Binary logistic regression was performed using on quartiles of total serum bilirubin using the last quartile (highest bilirubin value) as the reference. Data was adjusted for age and sex.

### Reduced levels of total serum bilirubin was confirmed in a validation cohort

The same i2b2 queries were performed at VCU as a validation cohort to confirm reduced levels of total serum bilirubin in a separate Caucasian population. Total serum bilirubin values were obtained from patients between January 1, 2007 to June 1, 2015 and for patients with more than one lab value, their results were averaged. The reference range for their laboratory is ≤1.30 mg/dL. Again, cases were 1:1 age- and sex-matched to controls, resulting in *n* = 233 subjects in the CD analysis and *n* = 124 subjects in the UC analysis. As with the PSU population, overall total serum bilirubin was lower in both CD (*P* <0.0001) and UC (*P* <0.0001) patients (Table 3). This same trend was seen when the data was stratified by age for most groups (5 out of 8 groups) and with the full data set (S3 Table). In addition, lower levels of serum bilirubin were independently associated with IBD by logistic regression analysis. The strongest association was demonstrated by patients with the lowest levels of bilirubin in the matched analysis for both CD (OR: 3.60 [95% CI: 2.19–5.99]) and UC (OR: 6.07 [95% CI: 3.01–12.75]) (Table 4) and the full data set (S4 Table).

**Table 3:** Comparison of total serum bilirubin between age- and sex-matched inflammatory bowel disease patients and controls at the Virginia Commonwealth University Medical Center cohort.

**Crohn’s Disease**					
**Age Group**	**CD (n)**	**CD Bilirubin (mg/dL)** **Median (IQR)**	**Controls (n)**	**Control Bilirubin (mg/dL)** **Median (IQR)**	***P*-value**
<20	4	0.30 (0.25–0.35)	4	0.45 (0.40–0.55)	0.053
20–39	110	0.40 (0.35–0.60)	110	0.60 (0.40–0.80)	**<0.0001**
40–59	80	0.43 (0.34–0.60)	80	0.54 (0.40–0.70)	**0.014**
≥60	39	0.43 (0.35–0.55)	39	0.50 (0.45–0.65)	**0.006**
Overall	233	0.40 (0.35–0.60)	233	0.55 (0.40–0.70)	**<0.0001**
**Ulcerative Colitis**					
**Age Group**	**UC (n)**	**UC Bilirubin (mg/dL)** **Median (IQR)**	**Controls (n)**	**Control Bilirubin (mg/dL)** **Median (IQR)**	***P*-value**
<20	3	0.50 (0.48–0.63)	3	0.40 (0.40–0.50)	0.376
20–39	61	0.45 (0.30–0.55)	61	0.60 (0.50–0.80)	**<0.0001**
40–59	37	0.50 (0.40–0.60)	37	0.60 (0.50–0.80)	**0.016**
≥60	23	0.50 (0.30–0.63)	23	0.65 (0.48–0.77)	0.071
Overall	124	0.50 (0.30–0.60)	124	0.60 (0.48–0.80)	**<0.0001**

Data collected from electronic medical records at Virginia Commonwealth University Medical Center from 2007–2015. Median and interquartile range (IQR) were calculated. *P*-value performed by Wilcoxen Rank Sum test.

**Table 4:** Odds of Crohn’s disease and ulcerative colitis by total serum bilirubin in the Virginia Commonwealth University Medical Center cohort.

Total Serum Bilirubin	Crohn’s Disease OR (95% CI)	Ulcerative Colitis OR (95% CI)
≤0.40 mg/dL	3.60 (2.19–5.99)	6.07 (3.01–12.75)
0.41–0.50 mg/dL	1.55 (0.85–2.81)	4.68 (2.17–10.45)
0.51–0.67 mg/dL	1.35 (0.76–2.41)	2.73 (1.22–6.21)
≥0.68 mg/dL	Reference	Reference

Binary logistic regression was performed using on quartiles of total serum bilirubin using the last quartile (highest bilirubin value) as the reference. Data was adjusted for age and sex.

## Discussion

Bilirubin is a potent antioxidant of lipid peroxidation products [4, 5]. In agreement with a previous report [3], lower levels of total serum bilirubin levels were found in CD patients compared to healthy controls. Correlating with these findings, previous studies described increased bilirubin levels within the gallbladder bile contents in patients with CD [11, 12]. Overall, these studies indicate that CD patients may have increased bilirubin metabolism that may result in reduced circulating total serum bilirubin. Similar to the CD findings, reduced total serum bilirubin levels were found in UC patients seen at Penn State Hershey Medical Center and at a validation cohort, Virginia Commonwealth University Medical Center. At PSU, UC patients with bilirubin levels ≤0.50 mg/dL were independently associated with disease relative to those with bilirubin levels ≥0.68 mg/dL (Table 4).

Functional defects in UGT1A1 result in unconjugated hyperbilirubinemia [13–16], which can be toxic to tissues if untreated and results in the characteristic jaundiced appearance. The UGT1A1*28 (rs8175347) polymorphism is a short microsatellite TA_(n)_ repeat in the TATA box upstream of the *UGT1A1* promoter. The wild-type allele consists of six TA repeats and the *28 polymorphism includes seven repeats. Approximately 10% of the Caucasian population is homozygous for the microsatellite polymorphism UGT1A1*28, which results in increased total serum bilirubin levels due to reduced transcriptional efficiency of *UGT1A1* and an overall 70% reduction in UGT1A1 enzymatic activity [17, 18]. Individuals that are homozygous for the UGT1A1*28 polymorphism have been shown to be at reduced risk (OR: 0.64 [95% CI: 0.42–0.98]) for developing CD [19]. This may be attributed to increased total serum bilirubin levels commonly seen in individuals homozygous for UGT1A1*28 which results in reduced transcriptional efficiency and glucuronidation activity, leading to reduced bilirubin metabolism and excretion.

Due to the decreased risk of CD associated with the *28/*28 genotype and increased serum bilirubin, further understanding how this pathway is regulated will help to elucidate the pharmacological potential. Increased *Ugt1a1* gene expression in the terminal ileum of DSS-induced colitis mice was previously described [20]. Activation of the PPARα-UGT axis and repression of FXR-FGF19 signaling was implicated in the Ugt1a1 response. This observation was confirmed in a small subset of human colonic biopsies from IBD patients and healthy controls [20]. Due to the retrospective design of our study, we were unable to determine how bilirubin is involved in IBD pathogenesis. However, previous studies have demonstrated that treatment of DSS-treated mice with bilirubin ameliorated the induction of colitis, inhibiting leukocyte migration and reduced the formation of ROS [21]. The results from our study are likely suggesting that low total serum bilirubin is inflammation-mediated rather than contributing to the disease process. This is not a phenomenon specific only to IBD as various other inflammatory diseases, including psoriasis vulgaris [22], nonalcoholic steatohepatitis and advanced fibrosis [23], rheumatoid arthritis [24, 25], and coronary artery disease [26, 27], demonstrated lower levels of total serum bilirubin. In particular, patients with moderate to severe inflammation due to nonalcoholic steatohepatitis were found to have significantly lower levels of total serum bilirubin compared to those with absent to mild inflammation, suggesting that bilirubin levels may correlate with inflammation [23]. A similar process may be occurring in IBD patients, although additional prospective studies are required.

As part of the metabolic pathway for bilirubin, intestinal β-glucuronidase deconjugates bilirubin in the gallbladder and intestines. In the human gastrointestinal tract, bacteria found among *Enterobacteriaceae* (i.e. *Escherichia coli*), *Ruminococcaceae*, *Lachnospiraceae*, and *Clostridiaceae* produce β-glucuronidase [28]. Interestingly, these bacteria are also implicated as potential drivers of IBD. In particular, *E*. *coli*, and specifically, the adherent-invasive strain, have been isolated from both CD and UC patients and display a more pronounced association with the mucosal lumen, suggesting that they may be directly involved in disease pathogenesis [29]. Similarly, *Ruminococcus* spp. are another group of bacteria that produce β-glucuronidase and are enriched in patients with IBD [30]. An abundance of β-glucuronidase-producing bacteria may alter bilirubin metabolism, resulting in the reduced levels of total serum bilirubin demonstrated in our study. Therefore, this relationship may be associated with bacterial dysbiosis and requires further investigation.

Limitations of this study should be considered when interpreting the data. Since a de-identified database was used to obtain bilirubin values, the disease severity was unknown at the time of blood draw. Variability in total serum bilirubin levels was seen for some patients with multiple lab tests. Therefore, the results were averaged for consistency since we were unable to stratify by potential confounding at the time of blood draw. Future prospective studies that evaluate total serum bilirubin at various stages of the disease process will aid in defining the role of total serum bilirubin in IBD. We were able to control for concomitant diagnoses that may influence bilirubin levels but could not address confounding medication usage or *UGT1A1* genotype at the time of blood collection. The inability to address other confounders is likely influencing the lack of significance in the ≥60 year old cohort in the PSU population and the differences in odds ratios between the PSU and VCU population. Caution should be taken when interpreting data for the <20 year old cohort due to small sample size and low power. Although, the data was not statistically significant, the trends were the same, with lower levels of total serum bilirubin in the cases compared to controls.

In summary, this study confirms other reports that individuals with CD had significantly lower levels of total serum bilirubin and extended these findings by demonstrating that UC patients also display reduced serum bilirubin levels. Specifically, our data demonstrated patients with the lowest levels of total serum bilirubin have the highest association with disease. Furthermore, our findings support the development of therapeutics that reduces oxidative stress in UC patients. In particular, IBD patients with reduced levels of the potent endogenous antioxidant, unconjugated bilirubin, may benefit from such therapies.

## Supporting information

S1 Table Comparison of total serum bilirubin between inflammatory bowel disease patients and controls at the Penn State Hershey Medical Center for the full data set. Data collected from electronic medical records at Hershey Medical Center from 2011–2014. Median and interquartile range (IQR) were calculated. *P*-value performed by Wilcoxen Rank Sum test.
(PDF)

S2 Table Odds of Crohn’s disease and ulcerative colitis by total serum bilirubin for the Penn State Hershey Medical Center full data set. Binary logistic regression was performed using on quartiles of total serum bilirubin using the last quartile (highest bilirubin value) as the reference. Data was adjusted for age and sex.
(PDF)

S3 Table Comparison of total serum bilirubin between inflammatory bowel disease patients and controls at the Virginia Commonwealth University Medical Center cohort. Data collected from electronic medical records at Virginia Commonwealth University Medical Center from 2007–2015. Median and interquartile range (IQR) were calculated. *P*-value performed by Wilcoxen Rank Sum test.
(PDF)

S4 Table Odds of Crohn’s disease and ulcerative colitis by total serum bilirubin in the Virginia Commonwealth University Medical Center cohort. Binary logistic regression was performed using on quartiles of total serum bilirubin using the last quartile (highest bilirubin value) as the reference. Data was adjusted for age and sex.
(PDF)
