# Supplementary material for: Reduced total serum bilirubin levels are associated with ulcerative colitis
Source: PLoS One. 2017 Jun 8;12(6):e0179267. doi: 10.1371/journal.pone.0179267 (PMC5464645; doi:10.1371/journal.pone.0179267)
Supplement: S1 Table — Data collected from electronic medical records at Hershey Medical Center from 2011–2014. Median and interquartile range (IQR) were calculated. P-value performed by Wilcoxen Rank Sum test. (PDF) [file pone.0179267.s001.pdf]

S1 Table. Comparison of Total Serum Bilirubin between Inflammatory Bowel Disease Patients and Controls at the Penn State Hershey Medical Center for the Full Data set

**Crohn's Disease**					
**Age Group**	**CD (n)**	**CD Bilirubin (mg/dL) Median (IQR)**	**Controls (n)**	**Control Bilirubin (mg/dL) Median (IQR)**	***P*-value**
<20	33	0.50 (0.40-0.65)	16	0.45 (0.38-0.78)	0.872
20-39	101	0.50 (0.40-0.65)	1,260	0.60 (0.47-0.80)	**<0.0001**
40-59	90	0.50 (0.40-0.70)	3,151	0.60 (0.50-0.77)	**0.0007**
≥60	56	0.58 (0.43-0.70)	1,742	0.60 (0.50-0.80)	**0.016**
Overall	280	0.50 (0.40-0.68)	6,169	0.60 (0.50-0.80)	**<0.0001**
**Ulcerative Colitis**					
**Age Group**	**UC (n)**	**UC Bilirubin (mg/dL) Median (IQR)**	**Controls (n)**	**Control Bilirubin (mg/dL) Median (IQR)**	***P*-value**
<20	9	0.35 (0.30-0.47)	16	0.45 (0.38-0.78)	0.110
20-39	63	0.50 (0.40-0.60)	1,260	0.60 (0.47-0.80)	**<0.0001**
40-59	72	0.55 (0.40-0.70)	3,151	0.60 (0.50-0.77)	**0.006**
≥60	63	0.60 (0.40-0.70)	1,742	0.60 (0.50-0.80)	**0.029**
Overall	207	0.50 (0.40-0.70)	6,169	0.60 (0.50-0.80)	**<0.0001**

Data collected from electronic medical records at Hershey Medical Center from 2011-2014. Median and interquartile range (IQR) were calculated. *P*-value performed by Wilcoxen Rank Sum test.
