# Supplementary material for: Reduced total serum bilirubin levels are associated with ulcerative colitis
Source: PLoS One. 2017 Jun 8;12(6):e0179267. doi: 10.1371/journal.pone.0179267 (PMC5464645; doi:10.1371/journal.pone.0179267)
Supplement: S3 Table — Data collected from electronic medical records at Virginia Commonwealth University Medical Center from 2007–2015. Median and interquartile range (IQR) were calculated. P-value performed by Wilcoxen Rank Sum test. (PDF) [file pone.0179267.s003.pdf]

S3 Table. Comparison of Total Serum Bilirubin between Inflammatory Bowel Disease Patients and Controls at the Virginia Commonwealth University Medical Center Cohort

**Crohn's Disease**					
**Age Group**	**CD (n)**	**CD Bilirubin (mg/dL) Median (IQR)**	**Controls (n)**	**Control Bilirubin (mg/dL) Median (IQR)**	***P*-value**
<20	36	0.34 (0.24-0.44)	4	0.45 (0.40-0.55)	0.113
20-39	130	0.40 (0.35-0.60)	465	0.50 (0.40-0.70)	**0.002**
40-59	80	0.43 (0.34-0.60)	737	0.50 (0.40-0.65)	**0.025**
≥60	43	0.40 (0.35-0.55)	359	0.50 (0.40-0.68)	**0.004**
Overall	289	0.40 (0.30-0.60)	1,565	0.50 (0.40-0.70)	**<0.0001**
**Ulcerative Colitis**					
**Age Group**	**UC (n)**	**UC Bilirubin (mg/dL) Median (IQR)**	**Controls (n)**	**Control Bilirubin (mg/dL) Median (IQR)**	***P*-value**
<20	17	0.45 (0.40-0.75)	4	0.45 (0.40-0.55)	0.964
20-39	62	0.45 (0.30-0.60)	465	0.50 (0.40-0.70)	**0.001**
40-59	37	0.50 (0.40-0.60)	737	0.50 (0.40-0.65)	0.461
≥60	26	0.50 (0.30-0.67)	359	0.50 (0.40-0.68)	0.729
Overall	142	0.50 (0.35-0.60)	1,565	0.50 (0.40-0.70)	**0.008**

Data collected from electronic medical records at Virginia Commonwealth University Medical Center from 2007-2015. Median and interquartile range (IQR) were calculated. *P*-value performed by Wilcoxen Rank Sum test.
