# Supplementary material for: Reduced total serum bilirubin levels are associated with ulcerative colitis
Source: PLoS One. 2017 Jun 8;12(6):e0179267. doi: 10.1371/journal.pone.0179267 (PMC5464645; doi:10.1371/journal.pone.0179267)
Supplement: S4 Table — Binary logistic regression was performed using on quartiles of total serum bilirubin using the last quartile (highest bilirubin value) as the reference. Data was adjusted for age and sex. (PDF) [file pone.0179267.s004.pdf]

S4 Table. Odds of Crohn's Disease and Ulcerative Colitis by Total Serum Bilirubin in the Virginia Commonwealth University Medical Center Cohort

Total Serum Bilirubin	Crohn's Disease OR (95% CI)	Ulcerative Colitis OR (95% CI)
≤0.40 mg/dL	2.56 (1.79-3.71)	1.95 (1.19-3.27)
0.41-0.50 mg/dL	1.57 (1.00-2.46)	2.35 (1.35-4.13)
0.51-0.67 mg/dL	1.26 (0.80-1.98)	1.42 (0.78-2.59)
≥0.68 mg/dL	Reference	Reference

Binary logistic regression was performed using on quartiles of total serum bilirubin using the last quartile (highest bilirubin value) as the reference. Data was adjusted for age and sex.
